# Supplementary material for: Meta-analysis of randomized controlled trials examining social comparison as a behaviour change technique across the behavioural sciences
Source: Nat Hum Behav. 2025 May 19;9(8):1595–612. doi: 10.1038/s41562-025-02209-2 (PMC12367546; doi:10.1038/s41562-025-02209-2)
Supplement: Supplementary file 1 — Supplementary Appendices A–E. [file 41562_2025_2209_MOESM1_ESM.pdf]

nature human behaviour

Supplementary information

https://doi.org/10.1038/s41562-025-02209-2

Meta-analysis of randomized controlled trials examining social comparison as a behaviour change technique across the behavioural sciences

In the format provided by the authors and unedited

Online supplementary material to the manuscript:

Meta-analysis of randomised controlled trials examining social comparison as a behaviour change technique across behavioural sciences

Thole H. Hoppen[1,*,#], PhD, Rieke M. Cuno[1,*], M.Sc., Janna Nelson[1], PhD, Frederike Lemmel[1], M.Sc., Pascal Schlechter[1], PhD, & Nexhmedin Morina[1,2], PhD

[1] Institute of Psychology, University of Münster, Münster, Germany
[2] Department of Psychology, New School for Social Research, New York, USA

*These authors contributed equally

#Corresponding author:
Thole H. Hoppen, Ph.D.
Institute of Psychology
University of Münster
Fliednerstr. 21
48149 Münster
Germany
Tel: +492518339415
Fax: +492518331331
e-Mail: thoppen@uni-muenster.de

Contents (hyperlinked)

Appendix A. Search string used for systematic literature search

Appendix B. Risk of bias assessment 2.0: Criteria per domain

Appendix C. Trial characteristics of randomised controlled trials excluded from the meta-analysis

Appendix D. Trial characteristics of randomised controlled trials included in the present meta-analysis

Appendix E. Ratings of indirectness following GRADE

Appendix A. Search string used for systematic literature search

Databases	Search Terms
MEDLINE and PsycINFO (via EBSCOhost)	(TI ("social compar*" OR upward comparison* OR downward comparison* OR "lateral comparison*") OR AB ("social compar*" OR upward comparison* OR downward comparison* OR "lateral comparison*") OR SU ("social compar*" OR upward comparison* OR downward comparison* OR "lateral comparison*"))
Web of Science	ALL=("social compar*" OR upward comparison* OR downward comparison* OR "lateral comparison*")

Note that the length of a search string/the number of search terms does not necessarily indicate the breadth of the search. The breadth of the search also depends on, a) which search terms (in the present work we used broad search terms), b) what is searched (in the present search strategy we searched in all-fields, which means that all sections of the full-text publication are searched for these terms), and c) how the search terms relate to one another in the search strategy (we only used “OR” connections and no “AND” connections, which means that any publication referring to one of the search terms in the full-text was retrieved in our search). The present search strategy resulted in more than 18,000 unique hits across the three databases searched (see Fig.1 in the main manuscript for more details). The search was conducted on Jan 2nd 2024 and covered all hits published from inception to Jan 2nd 2024.

Appendix B. Risk of bias assessment 2.0: Criteria per domain

**Domain**	**Sub-domains**
**Domain 1:**<br><br>Risk of bias arising from the randomization process.	• Was the allocation sequence random?<br>• Was the allocation sequence adequately concealed?<br>• Were there no differences between intervention groups at baseline suggesting no problem with the randomization process?
**Domain 2:**<br><br>Risk of bias due to deviations from the intended interventions.	• Were participants unaware of their assigned intervention during the trial?<br>• (if applicable) Were people delivering the interventions unaware of participants' assigned intervention during the trial?<br>• (if applicable) Were there no deviations from the intended intervention(s) (e.g., because of the context, way of delivery)?<br>• Was an appropriate analysis used?
**Domain 3:**<br><br>Risk of bias due to missing outcome data.	• Were data for the primary outcome available for all, or nearly all, participants randomized? If not, were ITT analyses/multiple imputations conducted?<br>• (if applicable) In case of missingness of outcome data, was the missingness random (i.e., equally distributed across arms)?<br>• (if applicable) Was there evidence that the result was not biased by missing outcome data?
**Domain 4:**<br><br>Risk of bias in measurement of the outcome.	• Was the method of measuring the primary outcome appropriate?<br>• Did the measurement or ascertainment of the outcome not differ between groups/arms?<br>• (if applicable) Were the outcome assessors unaware of the intervention received by study participants?<br>• (if applicable) Was the assessment of the outcome unlikely to have been influenced by knowledge of intervention received?
**Domain 5:**<br><br>Risk of bias in selection of the reported result.	• Was the trial analysed in accordance with a pre-specified plan that was finalized before outcome data were available for analysis?<br>• Was the numerical result being assessed unlikely to have been selected, on the basis of the results (e.g., from multiple outcome measurements within the outcome domain or from multiple analyses of the same data)?

Note. Each domain has a pre-defined algorithm on how to derive the judgment of a) low risk of bias vs. b) some concern vs. c) high risk of bias. This pre-defined algorithm is supplied by the Cochrane working group and can be retrieved here:
https://drive.google.com/file/d/1Q4Fk3HCuBRwIDWTGZa5oH11OdR4Gbhdo/view

Appendix C. Trial characteristics of randomised controlled trials excluded from the meta-analysis

Reference and study number if multiple studies were reported in publication	Description of intervention: SC-BCTs / the experimental group						Comparison group (comparison group category)	Outcome assessment		Basic study characteristics: country, statistical power, sample demographics, and risk of bias			
	SC-BCT applied in SC arm including SC dimension	SC direction (s)	SC standard (s)	Nr. of SC-BCT sessions	Target feedback level	Mode of presentation of SC standard; setting of SC induction	PCC arm, or ACC arm, or varying SC-BCT arm (for SC vs SC comparisons), or BCT bundle arm without SC-BCT (for add on comparisons)	Primary outcome (level of assessment); outcome category/behavioural domain; desired outcome vs. undesired outcome vs. mixed	Number of days between (first) SC-BCT and outcome assessment (i.e., short-term efficacy assessment)	Country of conduct; N	Sample description (sample type)	% females of total sample	Mean age (SD) total sample
Research question 1: SC-BCT as the stand-alone or main BCT vs. passive control conditions or active control conditions													
Ashraf et al. (2014)	SC of exam performance	Intended upward	Multiple persons	4	Individual	Passive SC via ranking; active SC with 1st and 2nd best performers regarding a) total score and b) improvement over time (written letter sent home); setting: training school	Feedback and temporal comparison (ACC)	Exam score (individual); performance; desired outcome	NR	Zambia; 308	Trainees of a health worker training program (population-based)	51%	27.02 (NR)
Brakel et al. (2011)	SC of coping with cancer (emotion vs. coping vs. combination tape)	Intended downward; intended upward; not restricted	Multiple persons	Not restricted; audio tape (20 min) freely available	No feedback	Three audio tapes covering interviews of cancer patients describing their coping experiences (emotion vs. coping vs. combination of both); setting: at home	Music: relaxing guitar music and a melodious voice without text (ACC)	Quality of life (individual); health; desired outcome	21	NL; 139	Cancer patients; initial or recurrent cancer of various types (physical health)	71%	51,94 (12.51)
Brakel et al. (2014)	SC of emotion coping	Not restricted	Multiple persons	Not restricted; audio tape (20 min) freely available	No feedback	Audio tape covering interviews of cancer patients describing their coping experiences individualised audio tape content depending on	Assessment only (PCC)	Quality of life (individual); health; desired outcome	60	NL; 150	Cancer patients; initial or recurrent cancer of various types (physical health)	81%	51.85 (11.41)

						patients' health status and SC sensitivity; setting: at home							
Buunk et al. (2012)	SC of coping with cancer	Intended upward	Multiple persons	NR	No feedback	Fellow cancer survivors (audio tape); setting: laboratory	Assessment only (PCC)	Quality of life (individual); health; desired outcome	14	NL; 226	Cancer patients (specific other)	65%	60 (NR)
Callery et al. (2021)	SC of energy usage	Not restricted	One group (average)	11	Group (apartment)	Low consumers compared with top 10th percentile of their group (top low consumers) and high consumers compared with average of their group; (digital written letter); setting: online	Assessment only (PCC)	Energy usage of last week; sustainability; undesired outcome	77	USA; 237	College Students (population-based)	NR	NR
Chabé-Ferret et al. (2019), Experiment 1	SC of water usage	Not restricted	One group (average)	11	Group (per farm)	SC (active) regarding water usage (digital written via SMS); setting: not restricted	Normative feedback regarding importance of saving water (ACC)	Water usage; sustainability; undesired outcome	77	France; 152	American farmers (other)	NR	54.41 (NR)
Delaval et al. (2017)	SC of statistic exercise performance	Not restricted	One group (average)	Not restricted	Individual	Score compared to the mean score of all first-year students (digital written letter); setting: online	Temporal comparison (ACC)	Number of successfully completed statistic exercises; performance; desired outcome	70	France; 123	Psychology students (student)	84%	18,93 (1.91)
Dillard et al. (2006)	SC of breast cancer risk	Downward SC with similar, worse off women	Multiple persons	1	Individual	Similar; worse off women (digital written letter); setting: laboratory	Intra-individual feedback via calculation of breast cancer risk (ACC)	Estimation of own breast cancer risk (individual); health; undesired outcome (i.e., decrease in overestimated risk targeted)	1	USA; 62	Women who overestimate own breast cancer risk (specific other)	100%	NR
Emmons & McCullough (2003)	SC of gratitude	Downward	NR	16	No feedback	Participants instructed to compare themselves mentally to others in general (verbal instruction); setting: laboratory	Gratitude condition and hassles condition (ACC)	Gratitude (individual); health; desired outcome	16	USA; 157	Undergraduate students (student)	NR	NR
Lew et al. (2007)	SC of various and self-chosen (non-appearance-related) dimensions	Intended downward	Multiple persons	4	No feedback	Fashion models (picture); setting: mixed – laboratory	Writing exercises (ACC)	Weight dissatisfaction (individual); health; undesired outcome (i.e., decrease in weight dissatisfaction targeted)	60	USA; 96	Female college students with higher levels of body dissatisfaction (specific other)	100%	19.09 (1.45)

Li et al. (2021), Study 1	SC of assignment progress (started vs. not started assignment yet)	Not restricted	Multiple persons	1	No feedback	Percentage of Peers in class/group who already started working on the assignment which is due in 4 days (digital written letter); setting: online	Assignment 3 is due in 4 days (ACC)	Participants who started with assignment in percent; health; desired outcome	NR	USA; 256	Undergraduate students in business analytics courses using excel (student)	41%	NR
Liu et al. (2022)	SC of room air conditioner energy use	Not restricted	One group (average)	Group 1: hourly = 90; Group 2: daily = 18	Household	SC of air conditioner energy usage of neighbour dorm rooms, hourly/daily feedback about air conditioner energy use (digital written letter); setting: at home	Assessment only (PCC)	Energy usage; sustainability; undesired outcome	18	China; 101	Students living in dormitories (student)	NR	NR
Verhaert & Van den Poel (2011)	SC of donation amount	Merged (not restricted; intended upward (more vs. less attainable))	NR	1	No feedback	SC of donor's donation amount "Another donor like you donated €x. You can also help us" with x being the recent, average, or maximum donation of another donor (written Mail); setting: online	No SC, only donation request: "Please help us by giving €x" (ACC)	Response rate to solicitation in %; the amount that was donated in €; revenue per appeal in €; service; desired outcome	60	Across Europe; 11 871	Prospects who never donated before, current contributors and donors who lapsed (population-based)	NR	NR
Research question 1: SC-BCT vs. (varying) SC-BCT													
Brakel et al. (2011)	SC of coping with cancer (emotion vs. coping vs. combination tape)	Intended downward; intended upward; not restricted	Multiple persons	Not restricted; audio tape (20 min) freely available	No feedback	Three audio tapes (one per condition) covering interviews of cancer patients describing their experiences (emotion vs. coping vs. combination of both); setting: at home	Three audio tapes (one per condition) covering interviews of cancer patients describing their experiences (emotion vs. coping vs. combination of both)	Quality of life (individual); health; desired outcome	21	NL; 139	Cancer patients; initial or recurrent cancer of various types (physical health)	71%	51,94 (12.51)
Brakel et al. (2012)	SC of coping with cancer	Not restricted	Multiple persons	Not restricted; intended upward; intended downward ; audio	Individual	Three audio tapes (one per condition) covering interviews with experts describing patients' experience (emotion vs. coping vs.	Three audio tapes (one per condition) covering interviews with experts describing	Quality of life (individual); health; desired outcome	60	NL; 154	Cancer patients; initial or recurrent cancer of various types (physical health)	68%	55 (11.11)

				tape (20 min) freely available		combination of both); one interview with cancer patient (content matched with participants); setting: at home	patients' experience (emotion vs. coping vs. combination of both); one interview with cancer patient (content matched with participants)					
Callery et al. (2021)	SC of energy usage	Not restricted	One group (average)	11	Group (apartment)	Participants within the 0 -25 (50 – 75) percentile of energy usage who were compared to the efficient (average) norm (digital written letter); setting: online	Participants within the 0 - 25 (75 – 100) percentile of energy usage who were compared to the efficient (average) norm	Energy usage of last week; sustainability; undesired outcome	77	USA; 237	College Students (population-based)	NR	NR
Singh et al. (2022)	SC of weight and weight loss	Not restricted	Multiple persons	1	No feedback	Screenshots of facebook posts of one female and one male user including supporting comments about weight loss (mixed online); setting: online	Screenshots of facebook posts of one female and one male user including supporting comments about weight loss	Goal commitment and healthy lifestyle intentions; health; desired outcome	0	Fiji; 804	Population, with a BMI > 30, social media use regular	54%	NR
Verhaert & Van den Poel (2011)	SC of donation amount	Not restricted; intended upward (more vs. less attainable)	NR	1	No feedback	SC of donor's donation amount "Another donor like you donated €x. You can also help us" with x being the recent, average, or maximum donation of another donor (written Mail); setting: online	SC of donor's donation amount "Another donor like you donated €x. You can also help us" with x being the recent, average, or maximum donation	Decision to donate; the amount that was donated in €; service; desired outcome	60	Across Europe; 11 871	Prospects who never donated before, current contributors and donors who lapsed (population-based)	NR	NR
Research question 2: BCT bundle with SC-BCT vs. BCT bundle without SC-BCT (i.e., efficacy of SC as an add-on intervention)												
Ho et al. (2022)	SC of distance walk, shop visits and money spent	Not restricted	Multiple persons	Not restricted	Individual	Leaderboard with other mall visitors and their points for distance walked and number of store visits	Coupons arm and badges arm (BCT bundles)	Points for distance walked and numbers of store visits; performance; desired outcome	56	China; 8 000	Mall visitors (population-based)	62%	33.9 (NR)

						(digital written letter); setting: shopping mall							
Preventive SC-BCTs (attempt of preventing undesired behaviour from occurring at a specific future occasion)													
Lewis et al. (2008)	SC of alcohol consumption	Not restricted	One group (average)	1	Individual	Normal number of drinks on 21st birthday (written; greeting cards); setting: online	Assessment only (PCC)	Number of alcoholic drinks on 21st Birthday; health; undesired outcome	14	USA; 187	College students, some of whom were abstainers at baseline, therefore preventive approach to some (student)	59%	21 (0)
Stamper et al. (2004)	SC of alcohol consumption	Not restricted	One group (average)	Not restricted	Individual	SC of the norm number of monthly drinks (mixed formats); setting: in class	Standard alcohol programming (ACC)	Drinking frequency (individual); health; undesired outcome	28	USA; 874	College students, some of whom were abstainers at baseline, therefore preventive approach to some (student)	61%	NR

Note. ACC = active control condition; BCT = behavioral change technique; NL = the Netherlands; NR = not reported (either not reported at all or in insufficient detail); PCC = passive control condition; SC = social comparison; SC-BCT add on = add on study in which SC was the only difference between two arms (i.e., the add on BCT); NR = not reported. Please find the references of these 14 publications in the reference list of the main manuscript (references# 49-62).

Appendix D. Trial characteristics of randomised controlled trials included in the present meta-analysis

Reference and study number if multiple studies were reported in publication	Description of intervention: SC-BCTs / the experimental group							Comparison group (comparison group category)	Outcome assessment		Basic study characteristics: country, statistical power, sample demographics, and risk of bias			
	SC-BCT applied in SC arm including SC dimension	SC-BCT as a stand-alone BCT (Yes vs. No)	SC direction (s)	SC standard (s)	Nr. of SC-BCT sessions	Target feedback level	Mode of presentation of SC standard; setting of SC induction	PCC arm, or ACC arm, or varying SC-BCT arm (for SC vs. SC comparisons), or BCT bundle arm without SC-BCT (for add on comparisons)	Primary outcome (level of assessment); outcome category; primary outcome = desired behaviour or undesired behaviour	Number of days between (first) SC-BCT and outcome assessment (i.e., short-term efficacy assessment)	Country of conduct; N	Sample description (sample type)	% of all participants identifying as females	Mean age (SD) of total sample
Research question 1: SC-BCT (as the stand-alone or the main behaviour change intervention) vs. passive control conditions or active control conditions														
Ambasta et al. (2023)	Social comparison of orders of routine laboratory tests, costs of orders, and lab free patients	No	Not restricted	One group (average) as well as multiple persons's performances	2	Individual	Average orders of routine laboratory tests, costs of orders, and lab free patients of fellow physicians (digital written letter); setting: online	Assessment only (PCC)	Number of ordered routine laboratory tests (individual); health; undesired behaviour	27	Canada; 179	Internists and general practitioners in clinical teaching units and hospitalist units (specific other)	NR	NR
Bator et al. (2019) Study 1	SC of electricity use	No	Not restricted	One group (average)	1	Household	Neighbours with similar sized apartments (written letter sent home); setting: at home	Face-to-face energy quiz (ACC)	Electricity usage (household); sustainability; undesired behaviour	28	USA; 450	Residents of apartments in urban environment (population-based)	NR	NR
Study 2	SC of electricity use	No	Not restricted	One group (average)	1	Household	Neighbours with similar sized apartments (written letter sent home); setting: at home	Assessment only (PCC)	Electricity usage (household); sustainability; undesired behaviour	77	USA; 1 035	Residents of apartments in urban environment (population-based)	NR	NR
Brent et al. (2020)	SC of water usage	No	Intended upward (10% goal)	One group (average)	1 or 2	Household	Neighbours with similar sized apartments (written letter[s] sent home); setting: at home	Assessment only (PCC)	Water usage (household); sustainability; undesired behaviour	60	USA; 21 151	Households (population-based)	NR	NR
Brülisauer et al. (2020)	SC of electricity usage	No	Not restricted	One group (average) or multiple persons	7	Individual	Entire residency (digital written letter); setting: online	Intra-individual feedback (ACC)	Electricity usage (individual);	37	Singapore; 398	Graduate students (student)	45%	NR

									sustainability; undesired behaviour					
Cadigan et al. (2019)	SC of alcohol consumption	No	Not restricted	One group (average)	NR	Individual	Same gender college students at same university (digital written letter); setting: at home	Information: effects of alcohol on the body (ACC)	Alcohol consumption (individual); health; undesired behaviour	1	USA; 130	Students who have been tailgating in the past month (student)	71%	21.01 (2.15)
Chapman et al. (2016)	SC of steps	No	Not restricted	NR	12	Individual	Fellow university staff members (digital written letter); setting: online	Tracking and trying to increase steps; message whether blog was completed (ACC)	Steps (individual); health; desired behaviour	14	USA; 64	University staff members (population-based)	94%	45 (NR)
Chen et al. (2010)	SC of movie ratings	No	Not restricted	One group (average)	1	Individual	Other users of MovieLens (digital written letter); setting: online	Intra-individual feedback: general information regarding own rating profile of one specific gee (ACC)	Movie ratings (individual); service; desired behaviour	28	USA; 398	Movielens users (population-based)	25%	NR
Chen et al. (2017)	SC of traffic violations	No	Not restricted	One group (average)	1	Group level	Owners of high status car; low status car; moderate status car; same car brand as participant (digital written letter); setting: online	Assessment only (PCC)	Traffic violations (individual); performance; undesired behaviour	30	China; 395 204	Car drivers with at least one traffic violation ticket in the first nine month of 2013 (population-based)	26%	39.59 (9.48)
Chen et al. (2023)	SC of electricity usage	No	Not restricted	One group (average) or two groups (average)	4	Team (group), team and room (groups), room (group)	Other teams (multiple rooms); other rooms within team and other teams; other rooms within team (digital written letter); setting: online	Assessment only (PCC)	Electricity usage (group); sustainability; undesired behaviour	32	Taiwan; 553	Undergraduate students living together in dormitory rooms (student)	NR	NR
Chevalier et al. (2025)	SC of sun protection behaviour	No	Not restricted	One person	1	No feedback	Sun protection behaviour (video); setting: online	Video on sleep hygiene (ACC)	Sun exposure and sun protection (individual); health; desired behaviour and undesired behaviour mixed	30	USA; 193	Young adult cancer survivors from survivorship clinics at Dana-Farber Cancer Institute (specific other)	66%	25.63 (4.93)
De Dominicis et al. (2019)	SC of electricity usage	No	Not restricted	One group (average)	NR	Household	Average electricity usage of similar households in neighbourhood (digital written letter); setting: at home	Assessment only (PCC)	Electricity usage (group); sustainability; undesired behaviour	810	USA; 390	Single-family households (population-based)	NR	NR
	SC of electricity usage	No	Not restricted	One group (average)	NR	Household	Average electricity usage of similar households in neighbourhood (digital written letter); setting: at home	Real intra-individual time feedback regarding last 3 minutes of energy consumption (ACC)	Electricity usage (group); sustainability; undesired behaviour	810	USA; 390	Single-family households (population-based)	NR	NR

Dohnke et al. (2018)	SC of healthy (fruits) and unhealthy (burger) diet	stand-alone BCT	Not restricted	Multiple persons	1	No feedback	Healthy or unhealthy eater of same sex (digital written letter); setting: school	Assessment only (PCC)	Consumption of sweets; (individual); health; undesired behaviour	28	Germany; 108	Students of 12 classes (8th class) of 6 schools (student)	39%	14.04 (0.64)
Earnhart & Ferraro (2021)	SC of waste water discharge	No	Not restricted	One group (average)	1	Facility	Water facility (written letter sent home); setting: facility	Assessment only (PCC)	Waste of water (facility); sustainability; undesired behaviour	90	USA; 255	Water facilities in Kansas (population-based)	NR	NR
Eyring & Narayanan (2018) Study 1	SC of activity level in course	No	Intended upward, not restricted	One group (average)	8	Individual	Average of other students (digital written letter); setting: online	Intra-individual feedback: information about progress for the course (ACC)	Activity level in course (individual); performance; desired behaviour	60	USA; 15 171	Student (student)	13%	32.03 (9.75)
Study 2	SC of grade	No	Intended upward, not restricted	One group (average)	8	Individual	Top quartile performing students (digital written letter); setting: online	Intra-individual feedback: information about progress for the course / temporal comparison (ACC)	Grade (individual); performance; desired behaviour	60	USA; 4 460	Student (student)	13%	30.62 (9.84)
Feizi & Khatabiroudi (2023)	SC of water consumption	No	Not restricted	Two groups (average)	6	(group) household	Average water consumption of neighbours and region (written letter sent home); setting: at home	Assessment only (PCC)	Water consumption (group); sustainability; undesired behaviour	360	Iran; 502	Households in water-critical city (population-based)	NR	NR
Ferraro & Price (2013)	SC of water usage	No	Not restricted	One group (average)	1	(group) household	Average neighbour (written letter sent home); setting: at home	Technical advice information only message (ACC)	Water usage (group); sustainability; undesired behaviour	60	USA; 106 669	Households (population-based)	NR	NR
	SC of water usage	No	Not restricted	One group (average)	1	(group) household	Average neighbour (written letter sent home); setting: at home	Assessment only (PCC)	Water usage (group); sustainability; undesired behaviour	60	USA; 106 669	Households (population-based)	NR	NR
Fleur et al. (2023) Study 1	SC of academic achievement	No	(Intended) upward	Multiple persons (9 students)	6	Individual	Average students with similar goals (picture); setting: online	Assessment of goal grade and outcomes (ACC)	Academic achievement (final grade; individual); performance; desired behaviour	49	Netherlands; 83	Students following the same course (student)	NR	NR
Study 2	SC of academic achievement	No	(Intended) upward	Multiple persons (9 students)	6	Individual	Average students with similar goals (picture); setting: online	Assessment of goal grade and outcomes (ACC)	Academic achievement (final grade; individual); performance; desired behaviour	49	Netherlands; 83	Students following the same course (student)	NR	NR
Gonçalves et al. (2018)	SC of basketball free throw	stand-alone BCT	Downward	One group (average)	1	Individual	Worse performing peers (i.e.; false feedback; always 20% worse than own score); (verbal); setting: at school	Intra-individual feedback (ACC)	Basketball free throw (individual); performance; desired behaviour	1	Brazil; 26	9-12 years-old pupils (student)	31%	9.63 (0.92)
Hamamura et al. (2023)	SC of smartphone usage	No	Not restricted	One group (average)	NR	Individual	Other students (written + image); setting: online	Intra-individual feedback concerning smartphone use per day via app (ACC)	Problematic smartphone use (individual); health; undesired behaviour	32	Japan; 305	Students from junior high school, high school, or technical College (student)	46 %	NR
Holladay et al. (2019)	SC of electricity usage in kWh	stand-alone BCT	Not restricted	One group (average)	1	Individual	Neighbors; (written letter sent home); setting: at home	Intra-individual feedback about own energy consumption in	Electricity usage in kWh (household); sustainability; undesired behaviour	60	USA; 100 548	Households (population-based)	NR	NR

								kWh via letter (ACC)						
Hong et al. (2022)	SC of writing progress	stand-alone BCT	Downward or upward (different arms)	Multiple persons	5	Individual	Worst writing examples (digital written letter); setting: at school	Classes as usual (ACC)	Writing progress (individual); performance; desired behaviour	60	Taiwan; 90	9th Grade Chinese Learners (student)	NR	14.47 (NR)
Hrozencik et al. (2023) study in Colorado	SC of water usage	No	Not restricted	Multiple groups (average)	3	Individual	Other well owners/operators (mixed formats); setting: at home	Assessment only (PCC)	Water usage (individual); sustainability; undesired behaviour	1095	USA; 863	Well owners/ operators (population-based)	NR	NR
study in Kansas	SC of water usage	No	Not restricted	Multiple groups (average)	2	Individual	Other wells (mixed formats); setting: at home	Assessment only (PCC)	Water usage (individual); sustainability; undesired behaviour	730	USA; 2 384	Well owners/ operators (population-based)	NR	NR
Kazukauskas et al. (2021)	SC of electricity usage	No	Not restricted	One group (average)	NR	Group (household)	Average neighbour apartments of similar size; (digital written letter); setting: at home	Intra-individual feedback via real time display smart meter (ACC)	Electricity usage (group); sustainability; undesired behaviour	60	Sweden; 525	Residents of newly built residential rental apartments (population-based)	NR	NR
Kim & Kaemingk (2021)	SC of electricity usage	No	Upward	One group (average)	1	Group (household)	Energy-efficient neighbour households; (written letter sent home); setting: at home	Assessment only (PCC)	Electricity usage (group); sustainability; undesired behaviour	60	Moldova; 127 760	Households (population-based)	NR	NR
Kitamura & Yamada (2025)	SC of time outside	No	Not restricted; upward	One group (median)	1	Individual	Other participants (written); setting: online	Information from emperor/ prime minister about Covid (ACC)	Time outside (individual); service; undesired behaviour	11	Japan; 2 868	Japanese population aged 20-59 years (population-based)	49 %	41.03 (10.76)
Klege et al. (2022)	SC of electricity usage	No	Intended upward	Multiple persons	20	Employees on same floor	Other floors in the same building; (digital written letter); setting: at work	Assessment only (PCC)	Electricity usage (floor); sustainability; undesired behaviour	60	South Africa; 988	Employees in government building (specific other)	NR	NR
Kollöffel & de Jong (2016)	SC of performance in trigonometry exercises	No	Not restricted	One group (average)	2	Individual	Other students; (digital written letter); setting: school	Criterion based intra-individual feedback; feedback (ACC)	Performance in trigonometry exercises (individual); performance; desired behaviour	60	Netherlands; 34	Students in secondary vocational engineering education (student)	0%	16.88 (0.95)
Krampen (1987)	SC of maths performance	No	Not restricted	NR	3	Individual	Other students; (written letters sent home); setting: at school	No intervention (PCC); subject matter-oriented comments (ACC); intra-individual feedback (ACC)	Maths performance (individual); performance; desired behaviour	60	Germany; 385	Students from secondary school (student)	49%	13.9 (1.4)
LaBrie et al. (2013)	SC of alcohol consumption	No	Intended upward	One group (average)	1	Individual	Students (level of specificity varies); (mixed formats); setting: online	Assessment only (PCC)	Alcohol consumption (individual); health; undesired behaviour	60	USA; 304	College students reporting at least on heavy episodic drinking event in last month (student)	57%	19.92 (1.3)
Lewis & Neighbours (2007)	SC of alcohol consumption	stand-alone BCT	Upward	One group (average)	1	Individual	Other students; (digital written letter); setting: laboratory	Assessment only (PCC)	Alcohol consumption (individual); health; undesired behaviour	60	USA; 122	College students (student)	45%	20.1 (1.8)
Lewis et al. (2007)	SC of alcohol consumption	stand-alone BCT	Upward	One group (average)	1	Individual	Other students; (digital written letter); setting: laboratory	Assessment only (PCC)	Alcohol consumption (individual); health; undesired behaviour	60	USA; 165	College students (student)	52%	18.53 (2.04)

Lewis et al. (2023)	SC of performance on motor sequence task	No	Downward	One group (average)	28	Individual	Average of 100 women/men between ages 50-75 (written); setting: laboratory	Assessment only (PCC); intra-individual feedback on response time (ACC)	Peak velocity on motor sequence task (individual); performance; desired behaviour	1	USA; 48	Sample from university and loval community (population-based)	69%	25.4 (5.2)
Liu & Lachman (2021)	SC of physical activity	No	Not restricted	Multiple persons	28	Individual	Other participants (digital written letter); setting: online	Intra-individual feedback regarding own daily step count (ACC)	Physical activity (individual); health; desired behaviour	60	USA; 56	Participants older than 60 (population-based)	75%	65.4 (5.31)
Lurbé et al. (2023)	SC of water usage	No	Not restricted	Two groups (average)	12	Household	Average & efficient; (written letter sent home); setting: at home	Assessment only (PCC)	Water usage (household); sustainability; undesired behaviour	60	USA; 10 154	Households (population-based)	NR	NR
Magnan et al. (2020)	SC of weekly exercise	stand-alone BCT	Upward	One group (average)	1	Individual	Other students of university; (digital written letter); setting: laboratory	Intra-individual feedback (ACC)	Weekly exercise (individual); health; desired behaviour	60	USA; 345	Undergraduate students (student)	69%	19.58 (0.19)
Mahler et al. (1999)	SC of diet and exercise compliance after coronary bypass surgery	No	Upward	Multiple persons	1	No feedback	Video; setting: hospital	Assessment only (PCC)	Diet and exercise compliance (individual); health; desired behaviour	60	USA; 215	Patients with first-time nonemergency CABG surgery (specific other)	14%	61.38 (8.35)
Mahler et al. (2010)	SC of sun-caused skin damage	No	Downward or upward	Multiple persons	1	Individual	Worse off other UV photographs (picture); setting: laboratory	Education (photo aging information) (ACC), assessment only (PCC)	Skin protection behaviour (individual); health; desired behaviour	60	USA; 126	College students (student)	77%	19.94 (2.36)
Mahler (2018)	SC of sun-caused skin damage	No	Downward or upward	Multiple persons	1	Individual	Worse off other UV photographs (picture); setting: laboratory	Education regardind photo aging (ACC)	Skin protection behaviour (individual); health; desired behaviour	60	USA; 223	Undergraduate students (student)	80%	20.97 (2.75)
	SC of sun-caused skin damage	No	Downward or upward	Multiple persons	1	Individual	Worse off other UV photographs (picture); setting: laboratory	Assessment only (PCC)	Skin protection behaviour (individual); health; desired behaviour	60	USA; 223	Undergraduate students (student)	80%	20.97
Max et al. (2016)	SC of virtual bike ride exercise	No	upward	Single person	9	No feedback	Virtual; superior bike ride partner (face to face); setting: laboratory	Solo virtual bike ride (ACC)	Exercise persistence (individual); health; desired behaviour	60	USA; 82	Persons interested in exercise (population-based)	56%	27.58 (9.51)
Meng et al. (2017)	SC of diet	No	Intended upward	Multiple persons	12	Individual	Other participants (digital written letter); setting: online	Intra-individual feedback (ACC)	Fruit and vegetable consumption (individual); health; desired behaviour	60	USA; 73	Undergraduate students (student)	67%	19.86 (1.65)
Michinov & Primois (2005)	SC of brainstorm contribution	No	Not restricted	Multiple persons	NR	Individual	Other participants (digital written letter); setting: online	Intra-individual feedback (ACC)	Brainstorm contribution (individual); performance; desired behaviour	60	France; 27	Adult learners (population-based)	44%	NR
Miller et al. (2016)	SC of alcohol consumption	stand-alone BCT	Not restricted	One group (average)	1	Individual	Same sex peers; (digital written letter); setting: online	Assessment only (PCC)	Alcohol consumption (individual); health; undesired behaviour	60	USA; 212	Psychology and speech students (student)	59%	19.89 (2.38)
Moseley et al. (2018)	SC of volunteering	stand-alone BCT	Intended upward	One group (average)	1	Individual	Top 10% or top 20% participants in the study; (digital written letter); setting: online	Intra-individual feedback regarding hours	Volunteering (individual); service; desired behaviour	60	England; 199	Students already volunteering (student)	70%	24.52 (NR)

								of volunteering (ACC)						
	SC of volunteering	stand-alone BCT	Not restricted	One group (average)	1	Individual	Participants in the study; (digital written letter); setting: online	Intra-individual feedback regarding hours of volunteering (ACC)	Volunteering (individual); service; desired behaviour	60	England; 140	Older and retired volunteers (population-based)	58%	63.27 (NR)
Mukai et al. (2022)	SC of electricity usage	No	Not restricted	Two groups (average)	12	Household	Other similar households (mixed formats); setting: at home	Intra-individual feedback regarding electricity usage from previous year (ACC)	Electricity usage (household); sustainability; undesired behaviour	60	Japan; 62 400	Households (population-based)	NR	NR
Mulgrew et al. (2018)	SC of exercise	No	Not restricted	Multiple persons	1	No feedback	Females engaging in exercise (video); setting: online	Control video (ACC)	Exercise behaviour (individual); health; desired behaviour	60	Australia; 256	Females (student)	100%	26.46 (5.5)
Myers & Souza (2020)	SC of electricity usage	No	Not restricted	Two groups (average)	13	Group (big: suite)	Student neighbours; (digital written letter); setting: at home	Assessment only (PCC)	Electricity usage (bedroom); sustainability; undesired behaviour	60	USA; 210	Undergraduate (student)	NR	NR
Neighbors et al. (2010)	SC of alcohol consumption	stand-alone BCT	Intended upward	One group (average)	1	Individual	Other students; (digital written letter); setting: online	Attention control (PCC)	Alcohol consumption (individual); health; undesired behaviour	60	USA; 321	College freshmen who reported at least one heavy-drinking episode in previous month (student)	58%	18.16 (0.6)
Neighbors et al. (2016)	SC of alcohol consumption	stand-alone BCT	Intended upward	One group (average)	1	Individual	Same sex students of same university; (digital written letter); setting: laboratory	Attention control (PCC)	Alcohol consumption (individual); health; undesired behaviour	60	USA; 623	Heavy drinkers = 4-5 drinks per occasion in last month (student)	53%	20.55 (1.7)
Neighbors et al. (2019)	SC of alcohol consumption	No	Intended upward	One group (average)	1	Individual	Students of the same university; (digital written letter); setting: laboratory	Assessment only (PCC)	Alcohol consumption (individual); health; undesired behaviour	60	USA; 188	Undergraduate heavy drinking college students (student)	54%	21.47 (2.04)
Otaki et al. (2022)	SC of water usage	No	Not restricted	One group (average)	10	Household	Households in the same condominium complex; (digital written letter); setting: at home	Assessment only (PCC)	Water usage (household); sustainability; undesired behaviour	60	Japan; 81	Households (population-based)	NR	NR
Qin & Chen (2021)	SC of electricity usage	stand-alone BCT	Not restricted	One group (average)	1	Group (dormitory)	Neighbour dormitories; (written letters sent home); setting: at home	Moral persuasion message with technical advice (ACC)	Electricity usage (group); sustainability; undesired behaviour	60	China; 574	Dormitories (student)	100%	NR
	SC of electricity usage	No	Not restricted	One group (average)	1	Group (dormitory)	Neighbour dormitories (written letters sent home); setting: at home	Assessment only (PCC)	Electricity usage (group); sustainability; undesired behaviour	60	China; 574	Dormitories (student)	100%	NR
Reiff et al. (2022)	SC of work performance /performed health screens	No	Intended upward	Multiple persons	5	Individual	Other physicians who also participated (digital written letter); setting: at work	Feedback via email, intra-individual comparison (ACC)	Work performance (individual); performance; desired behaviour	60	USA; 129	Physicians (specific other)	66%	NR
Steers et al. (2016)	SC of alcohol consumption	stand-alone BCT	Intended upward	One group (average)	1	Individual	Same-sex students of the same university (digital written letter); setting: online	Assessment-only (PCC)	Alcohol consumption (individual); health; undesired behaviour	60	USA; 112	College students who reported at least one heavy drinking episode in the past month (student)	82%	23.5 (4.96)

Van der Werff & Lee (2021)	SC of water usage	No	Not restricted	One group (average)	24	Household	Average of neighbourhood (digital written letter); setting: online	Tips and Tricks how to reduce residual waste (ACC)	Waste production (household); sustainability; undesired behaviour	60	Netherlands; 723	Households (population-based)	45%	50 (15)
Vasilaky et al. (2023)	SC of water usage	No	Not restricted	One group (average)	7	Household	Farmer group in the same village (written); setting: online	Assessment only (PCC)	Water usage in pumping hours (household); sustainability; undesired behaviour	14	India; 300	Rice farmer households (specific other)	NR	45.26 (12.94)
Villas-Boas et al. (2019)	SC of service participation	No	Not restricted	NR	1	Department	Other departments similar in size (digital written letter); setting: online	Call-to-service reminder email sent to department chairs/schools deans (ACC)	service participation (department); service; desired behaviour	60	USA; 1 126	Department and school employees (population-based)	NR	NR
Young & Neighbours (2019)	SC of alcohol consumption	No	Not restricted	One group (average)	1	Individual	Typical same-sex students at their university (digital written letter); setting: online	Attention control (PCC)	Alcohol consumption (individual); health; undesired behaviour	60	USA; 169	Heavy drinking undergraduates (student)	70%	21.02 (2.16)
Zhang et al. (2016)	SC of physical activity	No	Not restricted	Multiple persons	23	Individual	Other participants regarding performance in the programme (written letters sent home); setting: online	Solo use of SHAPE UP programme with individual incentives (ACC)	Physical activity (individual); health; desired behaviour	60	USA; 790	Graduate and professional students (student)	74%	25.2 (3.4)
Zhang et al. (2023)	SC of electricity use	No	Not restricted	Multiple persons	5	Group	Other peer students (written + image); setting: online	Assessment only (PCC)	Electricity consumption of dorms (group); sustainability; undesired behaviour	35	China; 192	College students living in dormitories (student)	55%	25.2 (3.4)
Zhong & Xia (2022)	SC of robotics course performance	No	Not restricted	Multiple persons	10	Pair feedback	Other peer students (verbal); setting: at school	Zero-sum coopetition with delayed reward intragroup cooperation intergroup competition with delayed reward (ACC)	Robotics course performance (individual); performance; desired behaviour	60	China; 80	Sixth graders (student)	40%	12 (NR)
Zhu et al. (2023)	SC of energy use	No	Not restricted	Multiple persons	5	Dorm (group)	efficient neighbours in school (top 20%), all neighbours in school, efficient neighbours in buildung (top 20%), neighbours in buildung, next-door neighbours, most efficient neighbours (top 20%) (written + image); setting: online	Assessment only (PCC)	Mean weekly energy consumption after intervention (kWh); sustainability; undesired behaviour	55	China; 480	Students from university in Zhuhai (student)	NR	NR
Zuckerman & Gal-Oz (2014) study 2	SC of step count	No	not restricted	Multiple persons	N R	Individual	Other participants (digital written letter); setting: online	Intra-individual feedback (ACC)	Physical activity (individual); health; desired behaviour	60	Israel; 59	Undergraduate (student)	75%	23.39 (1.4)
Research question 1: SC-BCT vs. (varying) SC-BCT														
Bogard et al. (2020)	SC of steps (less attainable upward vs. downward)	NA	Less attainable upward SC being	One group (average)	1	Individual	High performer (top 20%) (digital written letter); setting: online	Downward SC: being 39% better than high	Steps (individual); health; desired behaviour	1	USA; 121	Mechanical Turk users (population-based)	41%	36.1 (10.6)

			39% worse than high performers (upper 20%)					performer (upper 20%)						
Eyring & Narayanan (2018) study 1	SC of course activity level (intended upward vs. not restricted)	NA	Intended upward SC with top performance	One group (average)	8	Individual	Average of other students (digital written letter); setting: online	Feedback and not restricted SC with average performance	Course activity level (individual); performance; desired behaviour	60	USA; 15 171	Students (student)	13%	32.03 (9.75)
study 2	SC of grade (intended upward vs. not restricted)	NA	Intended upward SC with top performance	One group (average)	8	Individual	Top quartile performing students (digital written letter); setting: online	Feedback and not restricted SC with average performance	Grade (individual); performance; desired behaviour	60	USA; 4 460	Students (student)	13%	30.62 (9.84)
Hong et al. (2022)	SC of writing progress (upward vs. downward)	NA	Upward SC with best writing examples	Multiple persons	5	Individual	Anonymous students (digital written letter); setting: at school	Downward SC with worst writing examples	Writing progress (individual); performance; desired behaviour	60	Taiwan; 63	9th Grade Chinese Learners (student)	NR	14.47 (NR)
LaBrie et al. (2013)	SC of alcohol consumption (intended upward with higher peripheral similarity vs. intended upward with lower peripheral similarity)	NA	Intended upward SC with typical student of the same university	One group (average)	1	Individual	Other students; (mixed formats); setting: online	Web-BASICS-arm, intended upward SC with typical student	Alcohol consumption (individual); health; undesired behaviour	60	USA; 280	College students reporting at least on heavy episodic drinking event in last month (student)	57%	19.92 (1.3)
Lewis & Neighbours (2007)	SC of alcohol consumption (upward with higher peripheral similarity vs. upward with lower peripheral similarity)	NA	Upward SC with students of the same gender	One group (average)	1	Individual	Other students; (digital written letter); setting: laboratory	Upward SC with all students	Alcohol consumption, (individual); health; undesired behaviour	60	USA; 125	College students (student)	45%	20.1 (1.8)
Lewis et al. (2007)	SC of alcohol consumption (upward with higher peripheral similarity vs. upward with lower peripheral similarity)	NA	Upward SC with students of the same gender	One group (average)	1	Individual	Other students; (digital written letter); setting: laboratory	Upward SC with all students	Alcohol consumption, (individual); health; undesired behaviour	60	USA; 157	College students (student)	52%	18.53 (2.04)
Magnan et al. (2020)	SC of weekly exercise (more attainable upward vs. less	NA	Upward SC with the average	One group (average)	1	Individual	Other students of university; (digital written letter); setting: laboratory	Upward SC with the average student engaging	Weekly exercise (individual); health; desired behaviour	60	USA; 345	Undergraduate students (student)	69%	19.58 (0.19)

	attainable upward)		student engaging in 150 minutes of sport per week.					in 300 minutes of sport per week.						
Mahler et al. (1999)	SC of diet and exercise compliance after coronary bypass surgery (more attainable upward vs. less attainable upward)	NA	Upward SC with patients that cope successfully but describe difficulties and required effort	Multiple persons	1	No feedback	Other patients who had undergone coronary bypass surgery (video); setting: hospital room	Upward SC with patients who cope without mentioning difficulties	Light exercise (individual); health; desired behaviour	60	USA; 215	Patients with first-time nonemergency CABG surgery (specific other)	14%	61.38 (8.35)
Mahler et al. (2010)	SC of sun-caused skin damage (upward vs. downward)	NA	Upward SC with better off others	Multiple persons	1	Individual	Others UV photographs (picture); setting: laboratory	Downward SC (worse off others)	Skin protection behaviour (individual); health; desired behaviour	60	USA; 126	College students (student)	77%	19.94 (2.36)
Mahler (2018)	SC of sun-caused skin damage (upward vs. downward)	NA	Upward with better off others	Multiple persons	1	Individual	Others UV photographs (picture); setting: NR	Downward SC (worse off others)	Skin protection behaviour (individual); health; desired behaviour	60	USA; 223	Undergraduate students (student)	80%	20.97 (2.75)
Max et al. (2016)	SC of virtual bike ride exercise (more attainable upward vs. less attainable upward)	NA	Upward SC with superior bike ride partner who communicates fatigue from day 7	Single person	9	No feedback	Virtual; superior bike ride partner (face to face); setting: laboratory	Upward SC with superior bike ride partner who does not communicate fatigue	Exercise persistence (individual); health; desired behaviour	60	USA; 82	Persons interested in exercise (population-based)	56%	27.58 (9.51)
Meng et al. (2017)	SC of diet (more attainable upward vs. less attainable upward)	NA	Upward SC with increasingly healthier consuming peers	Multiple persons	12	Individual	3 other participants (high peripheral similarity; increasing & ideal consuming peers) (digital written letter); setting: online	Upward SC with ideal consuming peers	Fruit and vegetable consumption (individual); health; desired behaviour	60	USA; 73	Undergraduate students (student)	67%	19.86 (1.65)
Moseley et al. (2018), charity sample	SC of volunteering (more attainable intended upward vs. less attainable intended upward)	NA	Intended upward SC with median of top 20% volunteers	One group (average)	1	Individual	Top 10% or top 20% participants in the study; (digital written letter); setting: online	Intended upward SC with median of top 10% volunteers	Volunteering (individual); service; desired behaviour	60	England; 140	Charity sample: Older and retired volunteers (population-based)	58%	63.27 (NR)
and student sample	SC of volunteering (more attainable intended upward vs. less attainable intended upward)	NA	Intended upward SC with median of top 20%	One group (average)	1	Individual	Top 10% or top 20% participants in the study; (digital written letter); setting: online	Intended upward SC with median of top 10% volunteers	Volunteering (individual); service; desired behaviour	60	England; 199	Student sample: Students already volunteering (student)	70%	24.52 (NR)

			volunteers											
Patel et al. (2016)	SC of steps (intended upward vs. not restricted)	NA	Intended upward SC with 75th percentile of treatment group	One group (average)	13	Individual	Not restricted SC with 50th percentile of treatment group (mixed formats); setting: online	Daily and weekly feedback regarding step count	Steps (individual); health; desired behaviour	60	USA; 288	Employees & family members of university conducting study (population-based)	80%	41.3 (12)
Patel et al. (2020)	SC of steps (intended upward vs. not restricted)	NA	Intended upward SC with 75th percentile of treatment group	One group (average)	13	Individual	Not restricted SC with 50th percentile of treatment group (mixed formats); setting: online	daily and weekly feedback regarding step count	Steps (individual); health; desired behaviour	60	USA; 286	Employees & family members of university conducting study (population-based)	78%	37.17 (11.47)
Wulf et al. (2010)	SC of motor skills (upward vs. downward)	NA	Upward SC with average of 20% best performing participants	One group (average)	8	Individual	Other participants; (digital written letter); setting: laboratory	Downward SC with average of 20% worst performing participants	Motor skills transfer test (individual); performance; desired behaviour	60	USA; 28	University students (student)	43%	20.8 (3.53)
Research question 2: BCT bundle with SC-BCT vs. BCT bundle without SC-BCT (i.e., efficacy of SC as an add-on intervention)														
Leahey et al. (2020)	SC of weight loss	NA	Not restricted	Single person	38	Individual	Partners weight change (digital written letter); setting: online	Reduced Intensity Behavioural Weight Loss program (BCT bundle)	Weight (individual); health; undesired behaviour	60	USA; 278	Persons with a BMI between 30-40 (population-based)	76%	51.8 (NR)
	SC of weight loss	NA	Upward	Single person	38	Individual	Partners weight change (digital written letter); setting: online	Reduced Intensity Behavioural Weight Loss program (BCT bundle)	Weight loss (individual); health; desired behaviour	60	278	Persons with a BMI between 30-40 (population-based)	76%	51.8 (NR)
	SC of weight loss	NA	Upward		38	Individual	Partners weight change (digital written letter); setting: online	rBWL + Peer support arm (BCT bundle)	Weight loss (individual); health; desired behaviour	60	278	Persons with a BMI between 30-40 (population-based)	76%	51.8 (NR)
Lipkus & Klein (2006)	SC of colorectal cancer risk	NA	Lateral	One group (average)	1	Individual	Average of 100 women/men between ages 50-75 (mixed formats); setting: laboratory	General information about CRC (BCT bundle)	Colorectal cancer risk (individual); health; desired behaviour	60	USA; 160	Older age participants who were off schedule for having an FOBT (specific other)	66%	56 (NR)
	SC of colorectal cancer risk	NA	Intended upward	One group (average)	1	Individual	Average of 100 women/men between ages 50-75 (mixed formats); setting: laboratory	General information about CRC (BCT bundle)	Colorectal cancer risk (individual); health; desired behaviour	60	USA; 160	Older age participants who were off schedule for having an FOBT (specific other)	66%	56 (NR)

Meldrum et al. (2021)	SC of wildfire risk	NA	Not restricted	One group (average)	2	Household/ parcel	Neighbours; (written letter sent home); setting: at home	Feedback regarding own and community's wildfire risk (BCT bundle)	Wildfire risk (parcel); sustainability; desired behaviour	60	USA; 4 564	Households (population-based)	NR	NR
Spohrer et al. (2021	SC of health app use	NA	Upward	One group (average)	N R	Individual	Other health app users; (digital written letter); setting: online	Basic stress management elements (BCT bundle)	Health app use (individual); health; desired behaviour	60	USA; 138	Bachelor students (student)	53%	NR
Tomayko et al. (2020)	SC of 15 health-related habits	NA	Not restricted	Multiple persons	6	No feedback	Other participants (3 digital letters); setting: online	Individually completion of the Be Orange Challenge (spreadsheet to track 15 healthy habits daily) (BCT bundle)	Alcohol consumption (individual); health; desired behaviour	60	USA; 85	University employees (population-based)	87%	41.1 (18.6)
Visser et al. (2021)	SC of water usage	NA	Intended upward	NR	15	School	Water usage of other schools; (written letter sent home); setting: at school	Feedback of own usage across time via smart-metering, email reminders to safe water, posters with information and tips on water conservation in schools etc. without information regarding water usage of other schools (BCT bundle)	Water usage (school); sustainability; undesired behaviour	60	South Africa; 456 637	Primary and secondary school students (student)	NR	NR
Walters et al. (2009)	SC of alcohol consumption	NA	Intended upward	Two groups (average)	1	Individual	US. adults and student norms; (mixed formats); setting: laboratory	Single motivational interviewing session (BCT bundle)	Alcohol consumption (individual); health; undesired behaviour	60	USA; 143	Heavy drinking college students (student)	64%	19.8 (NR)
Zuckerman & Gal-Oz (2014) study 2	SC of step count	NA	not restricted	Multiple persons	N R	Individual	Other participants (digital written letter); setting: online	StepbyStep Application: Continuous measurement of steps, daily goal, real-time feedback and virtual rewards (BCT bundle)	Physical activity (individual); health; desired behaviour	60	Israel; 59	Undergraduate (student)	75%	23.39 (1.4)

Note. ACC = active control condition; BCT = behavioural change technique; PCC = passive control condition; SC = social comparison; SC-BCT add on = add on study in which SC was the only difference between two arms (i.e., SC as add on BCT); NA = not applicable; NR = not reported; CABG = coronary artery bypass graft; rBWL = reduced intensity behavioural weight loss treatment, CRC = colorectal cancer; FOBT = fecal occult blood test.

Appendix E. Ratings of indirectness following GRADE

Rating of indirectness for: Efficacy of social comparison as a behavioural change technique relative to passive control conditions at short-term

	Population		Intervention		Comparator		Outcome	
Reference		comments		comments		comments		comments
Ambasta et al. (2023)	Y		Y		Y		PY	multiple SC dimensions, including targeted behaviour
Bator et al. (2019) study 2	Y		Y		Y		Y	
Brent et al. (2020)	Y		Y		Y		Y	
Chen et al. (2017)	Y		Y		Y		Y	
Chen et al. (2023)	PN	students at dorms only	Y		Y		Y	
De Dominicis et al. (2019)	Y		Y		Y		Y	
Dohnke et al. (2018)	Y		Y		Y		PY	multiple SC dimensions, including targeted behaviour
Earnhart & Ferraro (2021)	Y	water facilities	Y		Y		Y	
Feizi & Khatabiroudi (2023)	Y		Y		Y		Y	
Ferraro & Price (2013)	Y		Y		Y		Y	
Hrozencik et al. (2023) study in Colorado	Y		Y		Y		Y	

Hrozencik et al. (2023) study in Kansas	Y		Y		Y		Y	
Kim & Kaemingk (2021)	Y		Y		Y		Y	
Klege et al. (2022)	Y		Y		Y		Y	
Krampen (1987)	Y		Y		Y		Y	
LaBrie et al. (2013)	Y		Y		Y		Y	
Lewis & Neighbors (2007)	Y	heavy drinking psychology students	Y		Y		Y	
Lewis et al. (2007)	Y	heavy drinking students	Y		Y		Y	
Lewis et al. (2023)	PN	18-40y participants for motoric improvement task	Y		Y		Y	
Lurbé et al. (2023)	Y		Y		Y		Y	
Mahler (2018)	PN	predominantly (80%) female students, 18-39y	Y		Y		Y	
Mahler et al. (1999)	PN	only 14% female participants	Y	video with social information highly relevant for patients	Y		PY	multiple SC dimensions, including targeted behaviour
Mahler et al. (2010)	PN	predominantly (77%) female students, 18-34y	Y		Y		Y	
Miller et al. (2016)	Y	drinking students	Y		Y		Y	
Myers & Souza (2020)	PN	students in dorms	Y		Y		Y	
Neighbors et al. (2010)	Y	heavy drinking students	Y		Y		Y	
Neighbors et al. (2016)	Y	heavy drinking students	Y		Y		Y	
Neighbors et al. (2019)	Y	heavy drinking students	Y		Y		Y	
Otaki et al. (2022)	Y		Y		Y		Y	
Qin & Chen (2021)	PN	students in dorms	Y		Y		Y	
Steers et al. (2016)	Y	heavy drinking students	Y		Y		Y	
Vasilaky et al. (2023)	Y	rice growing households	Y		Y		Y	

Young & Neighbors (2019)	Y	heavy drinking students	Y		Y		Y	
Zhang et al. (2023)	PN	students in dorms	Y		Y		Y	
Zhu et al. (2023) first-grade students	PN	students in dorms	Y		Y		Y	
Zhu et al. (2023) second-grade students	PN	students in dorms	Y		Y		Y	
Zhu et al. (2023) third-grade students	PN	students in dorms	Y		Y		Y	

Rating of indirectness following GRADE. N = no (i.e.., not direct); PN = probably not (i.e., probably not direct); PY = probably yes (i.e., probably direct); Y = yes (i.e., direct).

Rating of indirectness for: Efficacy of social comparison as a behavioural change technique relative to active control conditions at short-term

	Population		Intervention		Comparator		Outcome	
reference		comments		comments		comments		comments
Bator et al. (2019) study 1	Y		Y		Y		Y	
Brülisauer et al. (2020)	PN	students in dorms	Y		Y		Y	
Cadigan et al. (2019)	Y		Y		Y		Y	
Chapman et al. (2016)	PN	university staff members for population	Y		Y		Y	
Chen et al. (2010)	Y		Y		Y		Y	
Chevalier et al. (2025)	Y		Y		Y		PY	multiple SC dimensions, including targeted behaviour
De Dominicis et al. (2019)	PY	single-family households	Y		Y		Y	
Eyring & Narayanan (2018) study 1	PN	only 13% female	Y		Y		Y	
Eyring & Narayanan (2018) study 2	PN	only 13% female	Y		Y		Y	
Ferraro & Price (2013)	Y		Y		Y		Y	
Fleur et al. (2023) study 1	Y		Y		Y		Y	
Fleur et al. (2023) study 2	Y		Y		Y		Y	
Gonçalves et al. (2018)	Y		Y		Y		Y	
Hamamura et al. (2023)	Y		Y		Y		Y	

Holladay et al. (2019)	Y		Y		Y		Y	
Hong et al. (2022)	PN	students, but only 9th grades included	Y		Y		Y	
Kazukauskas et al. (2021)	PN	inhabitants of newly built homes only	Y		Y		Y	
Kitamura & Yamada (2025) intended downward; emperor arm	Y		Y		Y		Y	
Kitamura & Yamada (2025) intended downward; prime minister arm	Y		Y		Y		Y	
Kitamura & Yamada (2025) upward; emperor arm	Y		Y		Y		Y	
Kitamura & Yamada (2025) upward; prime minister arm	Y		Y		Y		Y	
Kollöffel & de Jong (2016)	PN	only male students	Y		Y		Y	
Krampen (1987)	Y		Y		Y		Y	
Lewis et al. (2023)	PN	18-40y participants for motoric improvement task	Y		Y		Y	
Liu & Lachman (2021)	Y		Y		Y		Y	
Magnan et al. (2020)	PY	students, who self-reported to be physically insufficiently active in the past week	Y		Y		Y	
Mahler (2018)	PN	predominantly (80%) female students, 18-39y	Y		Y		Y	
Mahler et al. (2010)	PN	predominantly (77%) female students, 18-34y	Y		Y		Y	

Max et al. (2016)	Y		Y		Y		PY	multiple SC dimensions, including targeted behaviour
Meng et al. (2017)	PN	undergraduate students	Y		Y		Y	
Michinov & Primois (2005)	Y		Y		Y		Y	
Moseley et al. (2018)	Y		Y		Y		Y	
Mukai et al. (2022)	Y		Y		Y		Y	
Mulgrew et al. (2018)			Y	video allowing for self-relevant SC on various dimensions but focusing on outcome-relevant dimension exercise	Y		PY	multiple SC dimensions, including targeted behaviour
Qin & Chen (2021)	PN	female students residing in student dorms	Y		Y		Y	
Reiff et al. (2022)	Y		Y		Y		Y	
van der Werff & Lee (2021)	Y		Y		Y		Y	
Villas-Boas et al. (2019)	PN	university staff of a large university (64 departments)	Y		Y		Y	
Ye et al. (2022)	PN	predominantly (>95%) male drivers	Y		Y		Y	
Zhang et al. (2016)	PN	students	Y		Y		Y	
Zhong & Xia (2022)	Y		Y		Y		Y	
Zuckerman & Gal-Oz (2014) study 2	PN	only communication students, predominantly female (75%)	Y		Y		Y	

Rating of indirectness following GRADE. N = no (i.e.., not direct); PN = probably not (i.e., probably not direct); PY = probably yes (i.e., probably direct); Y = yes (i.e., direct).

Rating of indirectness for: Efficacy of social comparison as a behavioural change technique relative to passive control conditions at long-term

	Population		Intervention		Comparator		Outcome	
reference		comments		comments		comments		comments
Ambasta et al. (2023)	Y		Y		Y		PY	multiple SC dimensions, including targeted behaviour
Bator et al. (2019) study 2	Y		Y		Y		Y	
Brent et al. (2020)	Y		Y		Y		Y	
Earnhart & Ferraro (2021)	Y	water facilities	Y		Y		Y	
Krampen (1987)	Y		Y		Y		Y	
LaBrie et al. (2013)	Y		Y		Y		Y	
Mahler et al. (1999)	PN	only 14% female participants	Y	video with social information highly relevant for patients	Y		PY	multiple SC dimensions, including targeted behaviour
Neighbors et al. (2010)	Y	heavy drinking students	Y		Y		Y	
Neighbors et al. (2016)	Y	heavy drinking students	Y		Y		Y	
Neighbors et al. (2019)	Y	heavy drinking students	Y		Y		Y	
Qin & Chen (2021)	PN	students in dorms	Y		Y		Y	
Vasilaky et al. (2023)	Y	rice growing households	Y		Y		Y	
Zhang et al. (2023)	PN	students in dorms	Y		Y		Y	

Rating of indirectness following GRADE. N = no (i.e.., not direct); PN = probably not (i.e., probably not direct); PY = probably yes (i.e., probably direct); Y = yes (i.e., direct).

Rating of indirectness for: Efficacy of social comparison as a behavioural change technique relative to active control conditions at long-term

	Population		Intervention		Comparator		Outcome	
reference		comments		comments		comments		comments
Cadigan et al. (2019)	Y		Y		Y		Y	
Krampen (1987)	Y		Y		Y		Y	
Liu & Lachman (2021)	Y		Y		Y		Y	
Max et al. (2016)	Y		Y		Y		PY	multiple SC dimensions, including targeted behaviour
Qin & Chen (2021)	PN	female students in dorms	Y		Y		Y	
Ye et al. (2022)	PN	predominantly (>95%) male drivers	Y		Y		Y	

Rating of indirectness following GRADE. N = no (i.e.., not direct); PN = probably not (i.e., probably not direct); PY = probably yes (i.e., probably direct); Y = yes (i.e., direct).

Rating of indirectness for: Efficacy of social comparison as an add-on behaviour change technique

	Population		Intervention		Comparator		Outcome	
reference		comments		comments		comments		comments
Leahey et al. (2020)	Y		Y		Y		PY	multiple SC dimensions, including targeted behaviour
Lipkus & Klein (2006)	Y		Y		Y		Y	
Meldrum et al. (2021)	Y		Y		Y		PY	information-seeking behaviour (i.e. visiting a website) that may allow for increased sustainability
Spohrer et al. (2021)	PN	only bachelor students included	Y		Y		Y	
Tomayko et al. (2020)	Y		Y		Y		PY	multiple SC dimensions, including targeted behaviour
Visser et al. (2021)	Y		Y		Y		Y	
Walters et al. (2009)	Y	heavy drinking students	Y		Y		Y	
Zuckerman & Gal-Oz (2014) study 2	PN	only communication students, predominantly female (75%)	Y		Y		Y	

Rating of indirectness following GRADE. N = no (i.e.., not direct); PN = probably not (i.e., probably not direct); PY = probably yes (i.e., probably direct); Y = yes (i.e., direct).

Rating of indirectness for: Efficacy of varying social comparison BCTs directly compared in the primary trials: Upward social comparison vs. downward social comparison

	Population		Intervention		Comparator		Outcome	
reference		comments		comments		comments		comments
Bogard et al. (2020); study 3b	Y		Y		Y		Y	
Hong et al. (2022)	PN	students, but only 9th grades included	Y		Y		Y	
Mahler (2018)	PN	predominantly (80%) female students, 18-39y	Y		Y		Y	
Mahler et al. (2010)	PN	predominantly (77%) female students, 18-34y	Y		Y		Y	
Wulf et al. (2010)	PN	students only	Y		Y		Y	

Rating of indirectness following GRADE. N = no (i.e.., not direct); PN = probably not (i.e., probably not direct); PY = probably yes (i.e., probably direct); Y = yes (i.e., direct).

Rating of indirectness for: Efficacy of varying social comparison BCTs directly compared in the primary trials: Upward social comparison vs. not restricted social comparison

	Population		Intervention		Comparator		Outcome	
reference		comments		comments		comments		comments
Brent et al. (2020)	Y		Y		Y		Y	
Eyring & Narayanan (2018) study 1	PN	only 13% female	Y		Y		Y	
Eyring & Narayanan (2018) study 2	PN	only 13% female	Y		Y		Y	
Moseley et al. (2018), charity sample	Y		Y		Y		Y	
Moseley et al. (2018), student sample	Y		Y		Y		Y	
Patel et al. (2016)	PN	university staff members and family	Y		Y		Y	
Patel et al. (2020)	PN	university staff members and family	Y		Y		Y	

Rating of indirectness following GRADE. N = no (i.e.., not direct); PN = probably not (i.e., probably not direct); PY = probably yes (i.e., probably direct); Y = yes (i.e., direct).

Rating of indirectness for: Efficacy of varying social comparison BCTs directly compared in the primary trials: More vs. less attainable social comparison at short-term

	Population		Intervention		Comparator		Outcome	
reference		comments		comments		comments		comments
LaBrie et al. (2013)	Y		Y		Y		Y	
Lewis & Neighbors (2007)	Y	heavy drinking psychology students	Y		Y		Y	
Lewis et al. (2007)	Y	heavy drinking students	Y		Y		Y	
Magnan et al. (2020)	PY	only students, but selected for insufficiently active	Y		Y		Y	
Mahler et al. (1999)	PN	only 14% female participants	Y	video with social information highly relevant for patients	Y		PY	multiple SC dimensions, including targeted behaviour
Max et al. (2016)	Y		Y		Y		PY	multiple SC dimensions, including targeted behaviour
Meng et al. (2017)	PN	undergraduate students	Y		Y		Y	
Moseley et al. (2018), charity sample	Y		Y		Y		Y	
Moseley et al. (2018), student sample	Y		Y		Y		Y	

Rating of indirectness following GRADE. N = no (i.e.., not direct); PN = probably not (i.e., probably not direct); PY = probably yes (i.e., probably direct); Y = yes (i.e., direct).

Rating of indirectness for: Efficacy of varying social comparison BCTs directly compared in the primary trials: More vs. less attainable social comparison at long-term

	Population		Intervention		Comparator		Outcome	
reference		comments		comments		comments		comments
Bogard et al. (2020); study 2	Y		Y		Y		Y	
LaBrie et al. (2013)	Y		Y		Y		Y	
Mahler et al. (1999)	PN	only 14% female participants	Y	video with social information highly relevant for patients	Y		PY	multiple SC dimensions, including targeted behaviour
Max et al. (2016)	Y		Y		Y		PY	multiple SC dimensions, including targeted behaviour

Rating of indirectness following GRADE. N = no (i.e.., not direct); PN = probably not (i.e., probably not direct); PY = probably yes (i.e., probably direct); Y = yes (i.e., direct).
