# Supplementary material for: Meta-analysis of randomized controlled trials examining social comparison as a behaviour change technique across the behavioural sciences
Source: Nat Hum Behav. 2025 May 19;9(8):1595–612. doi: 10.1038/s41562-025-02209-2 (PMC12367546; doi:10.1038/s41562-025-02209-2)
Supplement: Supplementary file 2 — Reporting Summary [file 41562_2025_2209_MOESM2_ESM.pdf]

nature portfolio

Corresponding author(s): Thole H. Hoppen, PhD

Last updated by author(s): Mar 12, 2025

Reporting Summary

Nature Portfolio wishes to improve the reproducibility of the work that we publish. This form provides structure for consistency and transparency in reporting. For further information on Nature Portfolio policies, see our Editorial Policies and the Editorial Policy Checklist.

Statistics

For all statistical analyses, confirm that the following items are present in the figure legend, table legend, main text, or Methods section.

n/a	Confirmed	
☐	☒	The exact sample size (n) for each experimental group/condition, given as a discrete number and unit of measurement
☐	☒	A statement on whether measurements were taken from distinct samples or whether the same sample was measured repeatedly
☐	☒	The statistical test(s) used AND whether they are one- or two-sided<br>*Only common tests should be described solely by name; describe more complex techniques in the Methods section.*
☒	☐	A description of all covariates tested
☒	☐	A description of any assumptions or corrections, such as tests of normality and adjustment for multiple comparisons
☐	☒	A full description of the statistical parameters including central tendency (e.g. means) or other basic estimates (e.g. regression coefficient) AND variation (e.g. standard deviation) or associated estimates of uncertainty (e.g. confidence intervals)
☐	☒	For null hypothesis testing, the test statistic (e.g. F, t, r) with confidence intervals, effect sizes, degrees of freedom and P value noted<br>*Give P values as exact values whenever suitable.*
☒	☐	For Bayesian analysis, information on the choice of priors and Markov chain Monte Carlo settings
☒	☐	For hierarchical and complex designs, identification of the appropriate level for tests and full reporting of outcomes
☐	☒	Estimates of effect sizes (e.g. Cohen's d, Pearson's r), indicating how they were calculated

Our web collection on statistics for biologists contains articles on many of the points above.

Software and code

Policy information about availability of computer code

Data collection	Data were extracted with Mircosoft Excel for Windows.
Data analysis	Statistical analyses were performed with the metafor package (v.3.4.0) in R (v.4.1.1). Risk of bias was evaluated with the Cochrane risk-of-bias tool (RoB 2.0). Visualisation of risk of bias assessments was done via the free online tool robvis (https://mcguinlu.shinyapps.io/robvis/). Certainty of evidence as assessed with GRADE criteria. All extracted data, the codebook, and analysis code are available on the Open Science Framework (OSF) at: https://osf.io/uwtbx/?view_only=e7fa20396d3c400e91607f8f81c77359

For manuscripts utilizing custom algorithms or software that are central to the research but not yet described in published literature, software must be made available to editors and reviewers. We strongly encourage code deposition in a community repository (e.g. GitHub). See the Nature Portfolio guidelines for submitting code & software for further information.

Data

Policy information about availability of data

All manuscripts must include a data availability statement. This statement should provide the following information, where applicable:

- Accession codes, unique identifiers, or web links for publicly available datasets
- A description of any restrictions on data availability
- For clinical datasets or third party data, please ensure that the statement adheres to our policy

This work was pre-registered with the PROSPERO database (PROSPERO-ID: CRD42022343154). The full data, codebook, and analysis code that support the findings of this study are available at the Open Science Framework at: https://osf.io/uwtbx/?view_only=e7fa20396d3c400e91607f8f81c77359

Research involving human participants, their data, or biological material

Policy information about studies with human participants or human data. See also policy information about sex, gender (identity/presentation), and sexual orientation and race, ethnicity and racism.

Reporting on sex and gender	Not applicable given that no new primary data was collected. Rather, available data was summarized in this meta-analysis. Included RCTs mostly concerned mixed-sex/diverse samples from the general population. We considered gender of pariticpants within a given sample within the GRADE assessment of certainty of evidence (i.e., assessment of directness in terms of population within studies).
Reporting on race, ethnicity, or other socially relevant groupings	Not applicable given that no new primary data was collected. We do report on the country of conduct for all included RCTs and state that most data in the field are from the US and other high-income countries. Most included RCTs did not report on race, ethnicity, or other socially relevant groupings making a comprehensive/meaningful summary of these variables across included trials infeasible We also report on the implications of most data stemming from the US and other high-income countries (i.e. limited generalisability of results) in the limitations (discussion section).
Population characteristics	Not applicable given that no new primary data were collected. Included RCTs mostly concerned large diverse samples from the general population. We considered population characteristics within the GRADE assessment of certainty of evidence (i.e., assessment of directness in terms of population within studies).
Recruitment	Not applicable given that no new primary data were collected. We do report in our overview tables concerning RCT characteristics (see Appendix D in the online supplementary materials) whether a general population vs. a specific population sample was recruited in the given RCT.
Ethics oversight	Not applicable given that no new primary data were collected.

Note that full information on the approval of the study protocol must also be provided in the manuscript.

Field-specific reporting

Please select the one below that is the best fit for your research. If you are not sure, read the appropriate sections before making your selection.

☐ Life sciences ☒ Behavioural & social sciences ☐ Ecological, evolutionary & environmental sciences

For a reference copy of the document with all sections, see nature.com/documents/nr-reporting-summary-flat.pdf

Behavioural & social sciences study design

All studies must disclose on these points even when the disclosure is negative.

Study description	Systematic review and meta-analysis with quantitative analysis of standardized mean differences
Research sample	Randomised controlled trials evaluating social comparison as a behaviour change technique. Studies had to meet all of the following inclusion criteria to be included in the present work: 1) RCT; 2) at least one arm investigated the efficacy of SC (as defined by Wood) as a BCT (as defined by Michie et al.) and was either a stand-alone or primary SC-BCT (research question 1) or an add-on SC-BCT (research question 2); 3) data for at least one behavioural outcome (e.g., electricity usage) were reported; 4) the outcome was assessed at least 24 hours after the induction of the (first) SC-BCT session to exclude experimental studies assessing only immediate reactions to social comparison; 5) available outcome data included at least ten participants per arm to exclude potential chance findings; and 6) data needed to be peer-reviewed. Withdrawn (i.e., retracted) publications were not included.
Sampling strategy	We formulated clear inclusion and exclusion criteria and then conducted a systematic literature search on MEDLINE, PsycINFO, and Web of Science up to Jan 2nd 2024. All studies that met the inclusion criteria and did not fulfill any exclusion criterion were included for analyses.
Data collection	Articles found through the abovementioned systematic search that matched our inclusion criteria were collected and data was extracted independently by two of the authors (THH & RMC).

Timing	This project was pre-registered on 17th July 2022. Conceptualization of this project started a few months earlier. Given that various steps of conduct took considerable time and given that we wanted to summarize a recent stage of the literature, the present work involved various search waves (with identical search strategy), which were added up and analyzed as a whole. The last search was performed on 2nd January 2024. This last search wave was added as part of the first (major) revision as part of the peer-review process. We worked continously on the present work since conceptualization (April 2022).
Data exclusions	Twelve RCTs did not report the relevant data in a usable format and did not reply to at least two data request emails and were thus excluded. Another two publications targeted preventing undesired behaviour from occurring in the future (i.e., alcohol consumption on a specific future occasion) and included various participants who did not engage in this undesired behaviour at baseline (i.e., abstainers) and were thus excluded. Lastly, another four publications reported RCTs that only assessed long-term cognitive or affective change following SC-BCTs but not behaviour change and were thus excluded. We provide basic characteristics of these 18 excluded RCTs (12+2+4) in Appendix C in the online supplementary materials.
Non-participation	Not applicable given that no new primary data were collected. In the present meta-analysis, we tried to minimize the risk of bias posed through attrition / data not missing at random in the primary studies (i.e., RCTs) by means of a) generally prioritizing ITT (intent-to-treat) data over completer data and b) by asking primary authors/corresponding authors of the primary work (i.e., RCTs) for ITT (rather than completer) data in our data request emails, which we sent when relevant data was missing. Missing outcome data of randomized participants was one of the five RoB2.0 domains and considered in the GRADE assessment of certainty of evidence.
Randomization	Not applicable given that no new primary data were collected. As mentioned above, the present work only included RCTs. "Risk of bias posed through shortcomings in the randomization process" was one of the five RoB2.0 domains evaluated and considered in the GRADE assessment of certainty of evidence.

Reporting for specific materials, systems and methods

We require information from authors about some types of materials, experimental systems and methods used in many studies. Here, indicate whether each material, system or method listed is relevant to your study. If you are not sure if a list item applies to your research, read the appropriate section before selecting a response.

Materials & experimental systems

n/a	Involved in the study
☒	☐ Antibodies
☒	☐ Eukaryotic cell lines
☒	☐ Palaeontology and archaeology
☒	☐ Animals and other organisms
☒	☐ Clinical data
☒	☐ Dual use research of concern
☒	☐ Plants

Methods

n/a	Involved in the study
☒	☐ ChIP-seq
☒	☐ Flow cytometry
☒	☐ MRI-based neuroimaging

Plants

Seed stocks	*Report on the source of all seed stocks or other plant material used. If applicable, state the seed stock centre and catalogue number. If plant specimens were collected from the field, describe the collection location, date and sampling procedures.*
Novel plant genotypes	*Describe the methods by which all novel plant genotypes were produced. This includes those generated by transgenic approaches, gene editing, chemical/radiation-based mutagenesis and hybridization. For transgenic lines, describe the transformation method, the number of independent lines analyzed and the generation upon which experiments were performed. For gene-edited lines, describe the editor used, the endogenous sequence targeted for editing, the targeting guide RNA sequence (if applicable) and how the editor was applied.*
Authentication	*Describe any authentication procedures for each seed stock used or novel genotype generated. Describe any experiments used to assess the effect of a mutation and, where applicable, how potential secondary effects (e.g. second site T-DNA insertions, mosiacism, off-target gene editing) were examined.*
